# Cbl-b deficiency provides protection against UVB-induced skin damage by modulating inflammatory gene signature

**DOI:** 10.1038/s41419-018-0858-5

**Published:** 2018-08-06

**Authors:** Tej Pratap Singh, Pablo A. Vieyra-Garcia, Karin Wagner, Josef Penninger, Peter Wolf

**Affiliations:** 1 Research Unit for Photodermatology, Department of Dermatology and Venereology, Medical University of Graz, Graz, Austria; 2 Center for Medical Research, Medical University of Graz, Graz, Austria; 3 Institute of Molecular Biotechnology (IMBA), Vienna, Austria; 4 Present Address: Inflammation Biology Section, Laboratory of Molecular Immunology, NIAID, NIH, Building 10, Room 11N112, 10 Center Drive, Bethesda, MD 20892 USA

## Abstract

Exposure of skin to ultraviolet (UV) radiation induces DNA damage, inflammation, and immune suppression that ultimately lead to skin cancer. However, some of the pathways that regulate these events are poorly understood. We exposed mice to UVB to study its early effects in the absence of Cbl-b, a known suppressor of antitumor immune response in the skin. Cbl-b^−/−^ mice were protected from UV-induced cell damage as shown by the lower number of cyclobutane pyrimidine dimers and sunburn cells in exposed skin compared to wild-type mice. Microarray data revealed that deficiency of Cbl-b resulted in differential expression of genes involved in apoptosis evasion, tumor suppression and cell survival in UV-exposed skin. After UVB, Cbl-b^−/−^ mice upregulated gene expression pattern associated with regulation of epidermal cell proliferation linked to Wnt signaling mediators and enzymes that relate to cell removal and tissue remodeling like MMP12. Additionally, the skin of Cbl-b^−/−^ mice was protected from chronic inflammatory responses and epidermal hyperplasia in a 4-weeks UVB treatment protocol. Overall, our results suggest a novel role for Cbl-b in regulating inflammation and physiologic clearance of damaged cells in response to UVB by modulating inflammatory gene signature.

## Introduction

Chronic exposure to ultraviolet (UV) irradiation is the leading cause of skin cancer including melanoma, squamous and basal cell carcinoma^1,2^. By the impingement of UV radiation, skin develops DNA photoproducts (PPs) such as cyclobutane pyrimidine dimers (CPDs) and 6–4PPs^3^. If such products are not eliminated by the repair mechanisms of the skin, mutations and subsequently cancers may arise^2^. The mechanisms that protect skin from the potential consequences of UV-induced DNA lesions involve active DNA repair by nucleotide excision, base excision and mismatch repair or as last resort induction of apoptosis and removal of cells with damages in their genome^3^. Additionally, genes involved in modulation of innate and adaptive immune responses including Cbl-b, may also play an important role in the immunomodulatory and carcinogenic effects of UVB exposure^3–5^. Cbl-b is a member of the mammalian Cbl family of proteins, a group of E3 ubiquitin ligases that consist of c-Cbl, Cbl-b, and Cbl-3. Cbl-b is a negative regulator of T-cell receptor signaling and its deficiency leads to spontaneous autoimmunity^6,7^. Moreover, Cbl-b is a modulator of many biological processes such as the induction of immune tolerance and antitumor immunity^8^. In addition, it has been demonstrated that Cbl-b-deficient mice developed fewer UVB-induced skin malignancies by spontaneously rejecting tumor cells in a CD8^+^ T-cell and NK-cell dependent manner^4,6,7^. Nevertheless, the role of Cbl-b in the early events that follow UVB exposure (relevant for the subsequent carcinogenic effect of sunlight) has not been evaluated. In the present work, we irradiated Cbl-b^−/−^ and wild-type (WT) mice with UVB to study the early responses of exposed skin in the absence of this ubiquitin ligase.

## Results

### Cbl-b-/- mice carry fewer UVB-induced SBCs and DNA PPs

**Fig. 1:**
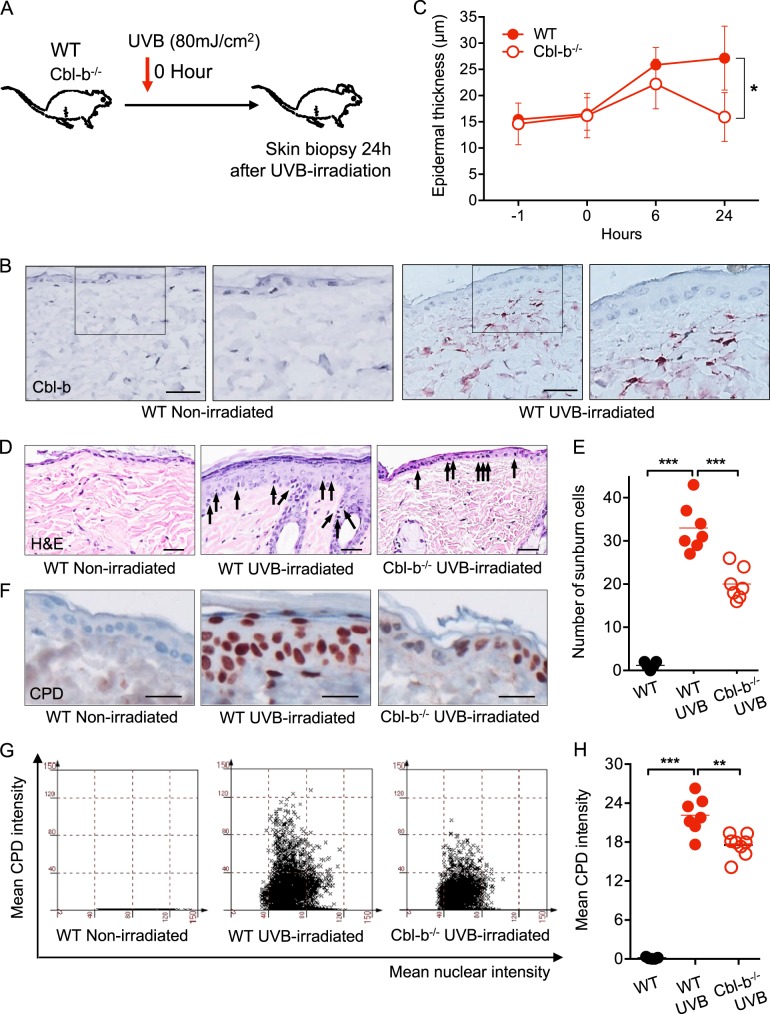
Cbl-b^−/−^ mice are protected from UVB-induced sunburn cell formation and DNA damage. **a** Schematic diagram showing the UVB irradiation protocol to induce short-term cytotoxicity. WT and Cbl-b^−/−^ mice were sacrificed at 24 h after the UVB exposure and dorsal skin samples were collected for SBC and CPD evaluation. **b** Cbl-b staining of dorsal skin sections of WT mice before and 24 h after UVB irradiation. Scale bar 50 μm. **c** Mean epidermal thickness during the first 24 h after UVB irradiation of WT and Cbl-b^−/−^ mice. Five mice were used per group, the experiment was done in three independent replicates. Representative data from three independent experiments. *n* = 5 mice per group. *P < 0.05; error bar is SD. **d** Representative H&E sections of dorsal skin to evaluate the number of SBCs. Arrows depict SBCs. **e** Averaged numbers of SBCs per field and their group mean in H&E sections of dorsal skin. *n* = 5–7 mice per group; ***P < 0.001. **f** Representative micrograph of CPD antibody-stained sections of dorsal skin to evaluate CPDs formation. **g** CPD antibody-stained sections of dorsal skin were analysed by tissue cell analysis. Representative plot of intensity of CPD- stained cells (*Y-axis*) and nuclear intensity (*X-axis*) for quantification of CPDs formation in epidermis of one mouse from each group. **h** Mean relative intensity of CPD positivity in epidermis of dorsal skin of individual mice. *n* = 5–7 mice per group; **P < 0.01, ***P < 0.001

Cbl-b participates in the rejection of UVB-induced tumor cells by enhancing cytotoxic immune responses mediated by tumor specific CD8^+^ cells^9^. We hypothesized that upon UVB irradiation, Cbl-b is highly upregulated and participates in the immunomodulatory effects of UVB in exposed skin. To evaluate the expression of Cbl-b in response to UVB, WT mice were irradiated with 80 mJ/cm^2^ of UVB (Fig. 1a). Samples of dorsal skin taken 24 h after UV exposure showed that cells mainly from the superficial dermis expressed cytoplasmic Cbl-b (Fig. 1b). Moreover, human skin irradiated with twice the minimal erythema dose of UVB also had Cbl-b^+^ cells infiltrating the superficial layer of the dermis (Suppl. Fig. 1). One of the early consequences of toxic UV exposure is the formation of SBCs. A SBC is a damaged epidermal cell undergoing apoptosis characterized by a pyknotic nucleus and condensed cytoplasm^10,11^. To investigate the role of Cbl-b in acute UVB-induced cytotoxicity, WT and Cbl-b^−/−^ UVB-irradiated mice were sacrificed at different time points to analyse epidermal thickness, SBCs, and CPDs. Cbl-b^−/−^ mice showed less epidermal thickening during the first 24 h after UVB exposure (Fig. 1c). We observed that 6 h after UVB irradiation, both WT and Cbl-b^−/−^ mice had a similar number of SBCs (Suppl. Fig. 2C). However, after 24 h, the skin of Cbl-b^−/−^ mice developed fewer SBCs compared to WT mice (i.e., 20 vs. 33 SBCs per microscopic field) (Fig. 1d, e). To evaluate the degree of DNA damage in UVB-irradiated skin, we carried out staining of thymine dimers, a specific type of CPD. Twenty-four hour after UVB irradiation; the skin of Cbl-b^−/−^ mice had significantly fewer CPDs compared to the skin of WT mice (Fig. 1f–h). To rule out differences of skin pigmentation between Cbl-b^−/−^ and WT mice (that may influence the number of SBCs and CPD in tissue) prior UVB exposure, we quantified pigmentation by noninvasive skin reflectance spectroscopy and the levels of melanin by Fontana-Masson staining and there was no influence of Cbl-b deficiency on skin pigmentation (Suppl. Fig. 2A, B). Together, these findings indicate that Cbl-b is an element of response to UVB expressed in cells of the dermis of mice and humans. Cbl-b plays a role in physiologic clearance of SBCs and its deficiency may provide protection from UVB exposure in a nonpigment-related manner.

### Cbl-b deficiency induces expression of epidermal growth-related genes and Wnt signaling in response to UVB irradiation

**Fig. 2:**
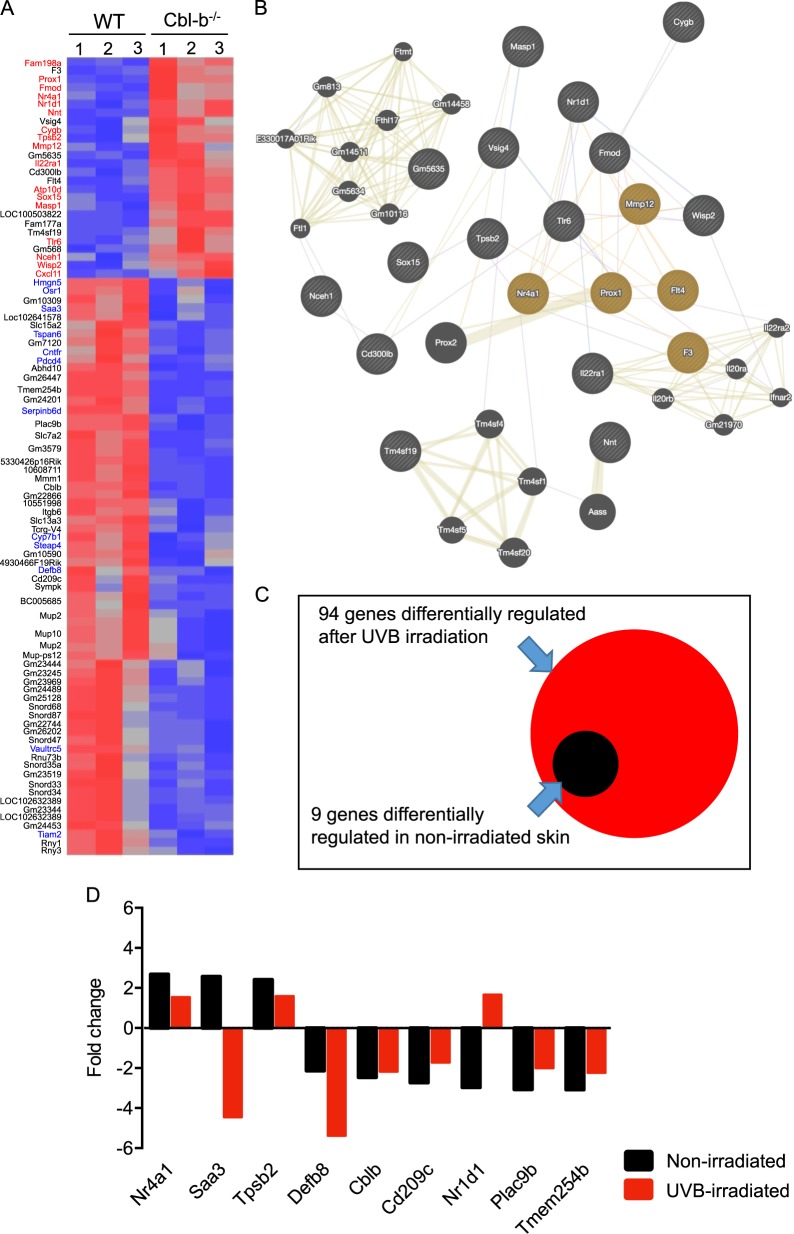
Gene expression analyzes on UVB-irradiated mice. WT and Cbl-b^−/−^ mice were sacrificed at 24 h after a single UVB exposure. Irradiated skin was excised to isolate RNA for microarray analysis (Affymetrix Mouse Gene 1.0 ST Array) and for immunostaining. **a** Heat map showing differentially expressed genes in Cbl-b^−/−^ compared to WT mice (*n* = 3 mice per group). Ninety-four genes were found deregulated as defined by a *p* value < 0.05 and ±1.5-fold change as cut off. Highest to lowest gene expression is depicted as red to blue color code in the heat map (Partek Genomics Suite 6.5). Gene names marked in red and blue are discussed in the Results section. **b** Gene interaction network of upregulated genes (large circles). A cluster of genes associated to positive regulation of epithelial cell proliferation was found (FDR = 0.0042, brown circles). A prediction of upregulated genes by shared domain (medium circles) or coexpression (small circles) was done with GeneMANIA webserver. Nodes are connected by co-localization (blue lines) and predicted/shared protein domains (brown lines). **c** Overlapping of differentially expressed genes in skin of Cbl-b^−/−^ vs. WT mice before (black) and after UVB irradiation (red). **d** Fold change comparison of nine genes with preexisting differential expression in nonirradiated and UVB-irradiated mice. Fold changes in Cbl-b^−/−^ vs. WT are shown in the waterfall plot

To gain insight of the gene expression profile that may be involved in protection against DNA damage induced by UVB irradiation in Cbl-b^−/−^ mice, we isolated RNA from skin samples taken before and 24 h after UVB irradiation of Cbl-b^−/−^ and WT mice to carry out microarray analyses. We established a cut off threshold of *p* value under 0.05 and a fold change level of ±1.5 to look for significant changes in gene expression. By doing so, we found 94 genes differentially regulated in Cbl-b^−/−^ vs. WT mice after UVB exposure (Fig. 2a). There was an overexpression of several genes involved in negative regulation of apoptosis (Wisp2, Fmod, Nceh1, Cygb, and Nnt)^12–16^, tumor suppression (Nr4a1, Sox15, and Msap1)^17,18^, oxidative-stress response (Atp10d, Cxcl11, Nnt, and Cygb)^12,16,19^, tissue remodeling (Mmp12)^20^, and cell survival (Tlr6, Nr1d1, Tpsb2, IL-22r1, Nr4a1, Prox1, and Fam198a)^21–23^ in UVB-exposed Cbl-b^−/−^ compared to WT mice. Many tumor-related (Tiam2, Tspan6, Hmgn5, Osr1, Pdcd4, Cntfr, and Vaultrc5)^24,25^ and inflammation-related (Saa3 and Defb8)^26^ genes were downregulated. A gene interaction network showed that within the upregulated genes after UVB irradiation, a cluster of five genes (Mmp12, Nr4a1, Prox1, Flt4, and F3) was directly associated to regulation of epithelial cell proliferation (Fig. 2b).

To understand whether the differentially expressed genes found in Cbl-b^−/−^ mice resulted from a direct response to UVB irradiation or a background alteration of Cbl-b deficiency, we carried out microarray analyses of the skin from nonirradiated Cbl-b^−/−^ and WT control mice (Fig. 2c). We observed that Cbl-b deficiency resulted in nine genes with differential expression as background, we plotted together the fold change values of expression from these genes comparing Cbl-b^−/−^ vs. WT mice of nonirradiated and UVB-irradiated animals (Fig. 2d). Three of these nine genes (Saa3, Defb8, and Nrd1) showed at least a twofold difference of expression after UVB irradiation between irradiated and nonirradiated animals, whereas the other six genes had minor fold change variations. Saa3 flipped from a 2.57-fold positive ratio to a 4.45-fold negative ratio; Nr1d1 flipped from a 2.97-fold negative to a positive 1.66-fold ratio; Defb8 decreased from a 2.14-fold negative ratio to a 5.38-fold negative ratio comparing nonirradiated vs. UVB-irradiated mice. We did not observe alterations in the expression of genes that are directly involved in DNA repair processes such as NER or BER between Cbl-b^−/−^ vs. WT mice after UVB irradiation, however, immunohistochemical stainings revealed that Cbl-b^−/−^ mice had higher expression of IL-10 24 h after exposure suggesting an increased immunomodulatory effect of UVB in these mice (Suppl. Fig. 3). Our data suggest that in response to UVB, cellular pathways involved in cell proliferation, activation, inflammation, invasion, and migration were greatly affected by the lack of Cbl-b (Suppl. Table 1).

**Fig. 3:**
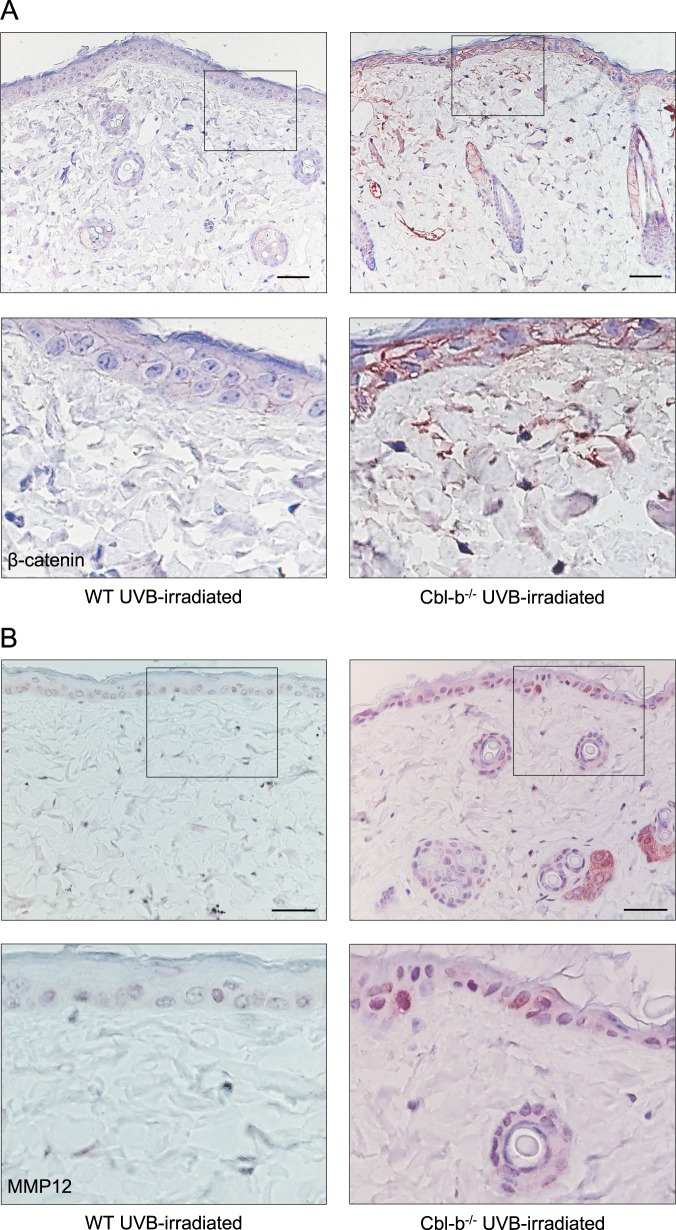
Cbl-b^−/−^ mice express β-catenin and MMP12 after UVB irradiation. Representative photographs from paraffin sections of dorsal skin of WT or Cbl-b^−/−^ mice at 24 h after UVB irradiation stained with β-catenin (**a**) and MMP12 (**b**). Marked squares of the upper panel are shown at higher magnification in the lower panel. Scale bar 50 μm

Among upregulated genes we found several members of the Wnt signaling pathway like Sox15, Wisp2, Nr4a1, Prox1, and Mmp12^27–29^. Therefore, we stained UVB-irradiated skin for β-catenin and MMP12 to evaluate Wnt activation and found a high number of β-catenin^+^ and MMP12^+^ cells in dermis and epidermis of Cbl-b^−/−^ compared to WT mice (Fig. 3a, b), suggesting an activation of Wnt signaling and a possible participation in tissue remodeling and removal of SBCs. Altogether, these results indicate that Cbl-b^−/−^ mice have differential transcriptional profile in UVB-irradiated skin that includes some genes from the background of Cbl-b deficiency, playing a role in regulation of cell proliferation and tumor/inflammation inhibition. Moreover, β-catenin activation suggests that Wnt signaling may crosstalk with Cbl-b as previous findings have suggested^30^.

### Cbl-b^−/−^ mice show lower degree of UVB-induced inflammation and epidermal hyperplasia

**Fig. 4:**
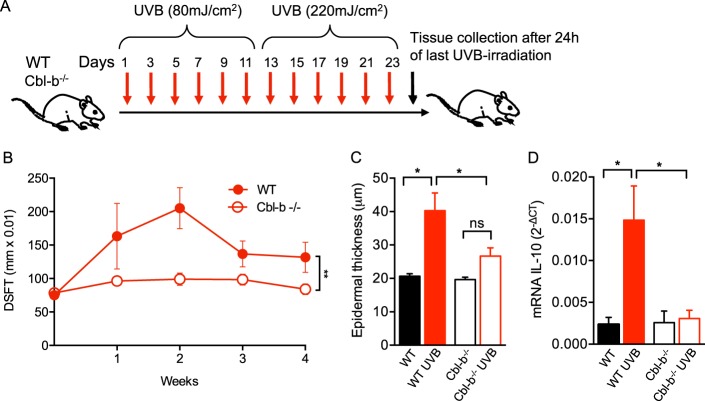
Cbl-b^−/−^ mice are protected from UVB-induced chronic inflammation. **a** Schematic diagram showing the UVB irradiation protocol to induce chronic inflammation. WT and Cbl-b^−/−^ mice were sacrificed at 24 h after the last UVB exposure. Dorsal skin was collected for analysis. **b** Mean double skin fold thickness (DSFT) of dorsal skin of WT and Cbl-b^−/−^ mice throughout 4 weeks of the UVB irradiation protocol. *n* = 5–7 mice per group. ^**^P < 0.01; error bar is SD. **c** Epidermal thickness of H&E-stained sections 24 h after last UVB exposure (at day 24). *n* = 5–7 mice per group. ^*^P < 0.05; error bar is SEM. **d** qRT-PCR analysis of IL-10 in dorsal skin. *n* = 5–7 mice per group. ^*^P < 0.05; error bar is SEM

Chronic inflammation is known to be a promotion factor in photocarcinogenesis. We tested whether deficiency of Cbl-b protects the skin from chronic inflammation. The dorsal skin of WT and Cbl-b^−/−^ mice was exposed to 6 doses of 80 mJ/cm^2^ and subsequent 6 doses of 220 mJ/cm^2^ during 23 days as described in Fig. 4a. Macroscopic double skin fold thickness (DSFT) and microscopic epidermal thickness were measured over time as readout of inflammatory response. Cbl-b^−/−^, but not WT mice were protected from chronic inflammation evidenced by a lower DSFT from the first week throughout the entire period of the UVB irradiation protocol (Fig. 4b). Epidermal thickening was reduced in UVB-irradiated Cbl-b^−/−^ mice compared to WT by the end of the UVB exposure protocol (Fig. 4c).

UVB-induced immunosuppression is crucial in the promotion of skin cancer. CD4^+^CD25^+^Foxp3^+^ regulatory T cells (Tregs) secreting IL-10 play an important role in the effect of UVB and participate in immune tolerance to UV-generated malignant cells^10^. After repetitive exposure to UVB we observed no significant changes in Tregs in the skin-draining lymph nodes of Cbl-b^−/−^ compared to WT mice (not shown). However, the expression of IL-10 was significantly reduced in the skin of Cbl-b^−/−^ mice (Fig. 4d). These results suggest that Cbl-b participates in sustaining chronic inflammation without interfering in the induction and/or recruitment of Tregs. Nonetheless, regulatory cytokines like IL-10 could be produced under Cbl-b regulation, thus, a lack of Cbl-b expression may abolish some of the immunomodulatory effects of UVB.

## Discussion

The regulatory function of Cbl-b has been explored in lymphocyte activation, autoimmunity and carcinogenesis. UVB-induced tumors are rejected in Cbl-b^−/−^ mice by an efficient activation of tumor specific cytotoxic CD8^+^ T cells^6^. In this study we addressed the immediate response to UVB of Cbl-b^−/−^ mice to understand the early events that may lead to the improved tumor immunity previously observed in these mice^6^. Irradiation of human skin showed infiltration of Cbl-b^+^ cells (Suppl. Fig. 1)suggesting that this ubiquitin ligase may have a relevant role in the effects of UVB such as carcinogenesis associated to excessive sun exposure. Cbl-b^+^ cells infiltrated the skin of WT mice after UVB exposure (Fig. 1b), possibly indirectly affecting removal of SBCs and CPDs in epidermis. In fact, the response to UVB-induced DNA damage can be modulated by inflammatory cytokines such as IL-10 and IL-18^31,32^. IL-10 staining 24 h after UVB exposure (Suppl. Fig. 3) demonstrated that Cbl-b deficiency resulted in a higher expression of this cytokine but after chronic exposure for up to 4 weeks and 12 sessions, qPCR results (Fig. 4d) showed a drastic drop in the expression of IL-10 in Cbl-b^−/−^ mice. Controversially, microarray results (Fig. 2a) and staining of XPC (not shown) did not indicate alterations in DNA repair machinery in Cbl-b^−/−^ mice although previous investigations have associated UVB-induced DNA damage with high levels of IL-10^33^. Interestingly, Cbl-b deficient effector T cells are resistant from Treg/IL-10 mediated suppression. Nevertheless, despite the immunosuppressive environment of the skin, Cbl-b deficiency promoted the clearance of SBCs.

After exposure to UVB, Cbl-b^−/−^ mice showed a reduced epidermal thickening compared to WT mice, suggesting a lower degree of overall cell injury (Fig. 1c). The quantification of CPDs and SBCs provides an accurate assessment of the damage in irradiated tissue and may predict the risk of tumor development. Indeed, SBCs are considered a surrogate marker of UV-induced DNA damage to the skin^34^. By using two different protocols of UVB exposure we studied the effects of Cbl-b deficiency on SBCs and CPDs formation and inflammation. We found that 24 h after exposure to a single dose of UVB, the skin of Cbl-b^−/−^ mice developed fewer SBCs and DNA dimers (Fig. 1c–h). The peak of UVB acute effects are regularly observed between 6 and 24 h, depending on UVB wavelength and dose^35,36^. SBC are visible as early as 30 min after irradiation and reach their highest in numbers within 24 h^37^, offering with the first 24 h after exposure a well delimited time-span to study immediate effects of UVB. The relevance of these early events post UVB irradiation are highlighted by studies in which the application of topical DNA repair enzymes reverts suppression in induction of contact hypersensitivity and protects from Langerhans cell depletion in UV-irradiated mice^38^. Moreover, DNA repair-deficient XP^39^ and IL-12p40^−/−^ mice exhibited an increased number of SBCs compared to WT after UVB irradiation^34,40^. Our results indicate that DNA PPs and SBCs are linked to Cbl-b expression, suggesting that Cbl-b may be involved directly or indirectly in controlling DNA repair mechanisms. Alternatively, the lack of Cbl-b may result in an enhanced removal of cells carrying DNA damage by an efficient cytotoxic activity of CD8^+^ and NK cells as demonstrated by previous results^6,7^. Moreover, we found no indication of a gains or losses in the mechanisms that cope with detoxification of ROS and DNA damage in Cbl-b^−/−^ mice since staining of 8-hydroxyguanosine, XPC and 6,4-PPs did not differ between WT vs. Cbl-b^−/−^ mice (not shown). Having said so, it is known that Cbl-b carries a UBA domain shared with the DNA repair protein Rad23, however, the functional role of the Cbl-b UBA domain is not known^41^.

The transcriptional profile of WT compared to Cbl-b^−/−^ mice after UVB irradiation showed 85 differentially regulated genes in addition to 9 genes with background differential expression in nonirradiated mice. Ubiquitin ligases like Cbl-b are known negative regulators of tyrosine kinase signaling. Their regulatory effect in cells of the immune system has been largely described^42^ and more recently, Cbl-b has been implicated in the maintenance of mammary stem cell phenotype involving a negative regulation of the AKT-mTOR pathway^43^. Our results suggest that Cbl-b deficiency may affect the transcriptional profile by shifting the activation of Wnt and tyrosine kinase signaling. The genes affected by UVB irradiation play a role in regulation of cellular processes like cell cycle, cell adhesion, invasion, and migration. It is known that Wnt signaling orchestrates DNA damage response through rescue of irradiated cells from apoptosis in a process regulated by c-Cbl targeting β-catenin^44,45^, a similar mechanism could also operate with Cbl-b. This observation can be supported by the presence of high number of β-catenin positive cells in UVB-irradiated Cbl-b^−/−^ mice (Fig. 3a). Although, β-catenin was upregulated after UVB irradiation together with other epidermal cell proliferation regulators, skin thickness of Cbl-b^−/−^ mice was lower compared to control WT mice after chronic exposure to UVB (Fig. 3b), suggesting that Cbl-b may limit the mechanisms that ameliorate UV damage to exposed cells and participate in the early events of phototoxicity and subsequent carcinogenesis. Moreover, metalloproteinases are enzymes that mediate photoaging and tissue remodeling^46^. Some metalloproteinases like MMP13 are negatively regulated by Cbl-b^47^. UVB-irradiated Cbl-b^−/−^ mice had an increase of MMP12 expression in cells of the epidermis (Fig. 3b), suggesting that upon UVB these mice may have a higher rate of tissue remodeling and elimination of SBC.

Excessive exposure to UVB is a known promotion factor of carcinogenesis. Our data indicate that Cbl-b deficiency protects from some of the effects of repetitive exposure to UVB as measured by lower cellular skin infiltration, swelling, and epidermal hyperplasia (Fig. 4b, c). Despite the low dose of UVB and the short period of irradiation used in our experiments (in comparison with studies of UVB tumor models^48^), IL-10 (a known mediator of UVB-induced immunosuppression^2,5,8,10^) was initially upregulated 24 h after a single exposure to UVB. Chronic exposure to UVB led to an increased expression of IL-10 in skin of WT mice, however, Cbl-b^−/−^ mice failed to upregulate this cytokine and showed lower levels of IL-10 (Fig. 4d). Animal models of photocarcinogenesis have demonstrated that regulatory T cells mediate a large proportion of the immunosuppressive effects of UVB with a peaks of infiltration in irradiated skin 7 days after initial exposure and subsequent normalization^49^. Nonetheless, our study was consistent with previous findings of unchanged Treg numbers in the skin after exposure of Cbl-b^−/−^ mice to UVB and a possible higher cytotoxic activity of CD8^+^ T cells by a decreased sensitivity to Treg-mediated immunosuppression^6^. Whether the origin of the observed protective Cbl-b^−/−^ phenotype is due to the decreased DNA damage in the epidermis, an autocrine effect or via interaction with the altered immune system in these mice remains to be determined.

In summary, UVB irradiation in Cbl-b deficient mice leads to a lower number of DNA PPs and SBCs, higher expression of Wnt signaling mediators like β-catenin and a differential gene expression signature enriched with cell proliferation regulators compared to WT control mice. This suggests that Cbl-b is involved in the immediate response to UVB cytotoxicity. Thus, if cells can indeed better tolerate UVB and survive in the absence of Cbl-b (without harboring internal damage), then our findings may open new avenues to target this gene in novel sun protection strategies.

## Materials and methods

### Animals

BL6 WT and Cbl-b^−/−^ mice were obtained from the Institute of Molecular Biotechnology (IMBA) Vienna, Austria and held in our facility at the Medical University of Graz. All animals were maintained with alternating 12 h light and dark cycles, as well as controlled temperature and humidity in our facilities. Mice were shaved on the dorsal skin 1 day prior experimental procedures. All animal procedures were approved by the Federal Ministry of Science and Research, Austrian Government through protocol no. BMWF-66.010/0019-II/3b/2011. All methods were performed in accordance with the relevant guidelines and regulations.

### UVB irradiation of animals

Mice were exposed to a single dose of 80 mJ/cm^2^ of UVB. A Waldmann UV236B irradiation system equipped with two fluorescent CF-L 36 W/UV6 light tubes (emission range, 280–360 nm; peak, 324 nm; Waldmann Medizintechnik, Villingen-Schwenningen, Germany) was used for UVB irradiation, at a mean irradiance of 2.20 mW/cm^2^ at a distance of 15 cm and the irradiance of exposure was monitored by a calibrated Waldmann photometer. The applied UVB dose corresponded to approximately one minimal erythema dose, as determined by exposure to a series of UVB doses at the increments by factor of 1.4, as previously described^50^. To study the chronic effects, mice were exposed to repetitive UVB doses on alternate days for 23 days at a dose of 80 mJ/cm^2^ for 11 days and then at a dose of 220 mJ/cm^2^ from day 12 to 23 by using the same irradiation system. Skin inflammation was monitored by measuring the DSFT of dorsal skin with an engineer’s micrometer (Mitutoyo Corporation) and mice were killed 6 or 24 h after last UVB exposure for tissue collection. Noninvasive skin pigmentation was determined by skin reflectance spectroscopy on shaved dorsal skin with a DermaSpectometer (Cortex Technologies, DK).

### Humans skin samples

Paraffin-embedded materials were available for immunohistochemical stainings from a previous clinical study. In accordance with the study protocol (IRB approval number: 15–129 ex 93/94) healthy volunteers had been irradiated on their buttocks with solar simulated UV radiation equivalent to two times of the minimal erythema dose and skin biopsies had been taken 24 h after exposure.

### Histological evaluation

Epidermal hyperplasia was assessed on H&E-stained sections of dorsal skin by measuring the histologic thickness of the epidermis. SBCs were counted in the interfollicular epidermis of H&E-stained dorsal skin sections in at least 10 random fields (at a final magnification 20×). All measurements were performed in a blinded manner. Photographic images were acquired by using a DP71 digital camera (Olympus) attached to an Olympus BX51 microscope.

### Immunohistochemical and image analysis

Skin sections of mice were pretreated with EDTA at pH 8 and then incubated with antithymine dimer (clone KTM53; Kamiya Biomedical Co, Seattle, WA) (1:2000), anti-MMP12 (clone EP1261Y; Abcam ab52897) anti-IL-10 (clone JES5-2A5; Abcam ab189392) or anti-β-catenin monoclonal antibody (clone E247; Abcam, Oxford UK). Sections of human and mice skin were stained with anti Cbl-b (clone 246C5a; Abcam ab54362). The Dako K 5003 detection system (Dako, Glostrup, Denmark) was used for visualization according to the manufacturer’s instructions. Melanin determination was performed by standard Fontana-Masson staining (Ab1506669, Abcam, Oxford, UK) according to manufacturer instructions. To quantify CPDs, antibody-stained tissue sections were scanned on TissueFAXS system by TissueFAXS cell tissue analysis software (TissueGnostics GmbH, Vienna, Austria) and the epidermis was electronically dissected with ImageJ software for intensity measurement by TissueQuest software (TissueGnostics GmbH, Vienna, Austria).

### Quantitative RT-PCR

RNA was extracted from dorsal skin tissue using QIAGEN fibrous mini kit (QIAGEN) and cDNA was made by using First strand cDNA synthesis kit (Roche). Quantitative RT-PCR for IL-10 was performed on an Applied Biosystems 7900HT system by using RT. SYBR Green/ROX qPCR Master Mix (SABiosciences). The 2-ΔCt method was used to normalize the transcript to GAPDH.

### Microarray analysis

The Affymetrix Gene Chip Microarray platform was used for identifying genes that are transcriptionally influenced. We processed three biological replicates of dorsal skin RNA samples collected from WT and Cbl-b^−/−^ mice before and 24 h after exposure to a single dose of 80 mJ/cm^2^ UVB on the Affymetrix Mouse Gene 1.0 ST Array (Affymetrix, Santa Clara, CA, USA) to determine the transcriptional profile. Total RNA was extracted as described above and checked for quality on the BioAnalyzer BA2100 (Agilent, Foster City, CA). For amplification, 400 ng of the total RNA was used with the Ambion Whole Transcript Expression Kit for Affymetrix GeneChip, Whole Transcript Expression Arrays (Life Technologies; Carlsbad, California). The first strand cDNA was created according to the manufacturer protocol, which then synthesized the second strand cDNA. During the following in vitro transcription, complementary RNA (cRNA) was generated. After purification, a second cycle of first strand cDNA synthesis was performed implementing dUTPs for fragmentation. RNaseH hydrolyzed the cRNA, followed by an enzymatic fragmentation and biotin-labeling (Affymetrix GeneChip Whole Transcript Terminal Labeling and Hybridization for use with Ambion Whole Transcript; Affymetrix, Santa Clara, CA, USA). We hybridized the fragmented samples overnight rotating the arrays at 60 rpm. After washing at the Affymetrix Genechip fluidics station 450, protocol FS450_0007 (Affymetrix GeneChip^®^ HT hybridization, Wash, and Stain Kit; Affymetrix, Santa Clara, CA, USA) the arrays were scanned with the Affymetrix Scanner GCS3000, AGCC (Command Console software AGCC 3.1.1) with default analysis settings for generation of CEL-files. The arrays were evaluated based on the internal array controls using Affymetrix Genexpression Console (1.1.2). Gene interaction analysis was done using the GeneMANIA webserver^51^. CEL-files were imported into Partek Genomic Suite v6.6 software (Partek Inc., St Louis, MO) for performing robust multichip average normalization including background correction, quantile normalization across all arrays, and median polished summarization based on log transformed expression values. A fold change of ±1.5 in gene expression was considered as differentially relevant. To gain insight into functional processes we used Ingenuity Pathway Analysis (IPA; Qiagen, Redwood City, CA). Differentially expressed genes in Cbl-b^−/−^ vs. WT mice before and after UVB irradiation were compared and plotted in a waterfall graph. Raw data is available on the Gene Expression Omnibus (GEO) database with the dataset number GSE79073.

### Statistical analysis

Data were expressed as mean ± SEM. Statistical differences among experimental groups were determined by using two-tailed unpaired *t* test or one-way ANOVA (for microarray analysis). Statistical significance was set at *P* < 0.05.

## Electronic supplementary material

Supplementary figure 1

Supplementary figure 2

Supplementary figure 3

Supplementary table 1: Gene co-expression analyses
