# Supplementary material for: Cbl-b deficiency provides protection against UVB-induced skin damage by modulating inflammatory gene signature
Source: Cell Death Dis. 2018 Aug 6;9(8):835. doi: 10.1038/s41419-018-0858-5 (PMC6079082; doi:10.1038/s41419-018-0858-5)
Supplement: Supplementary file 1 — Supplementary figure 1 [file 41419_2018_858_MOESM1_ESM.pdf]

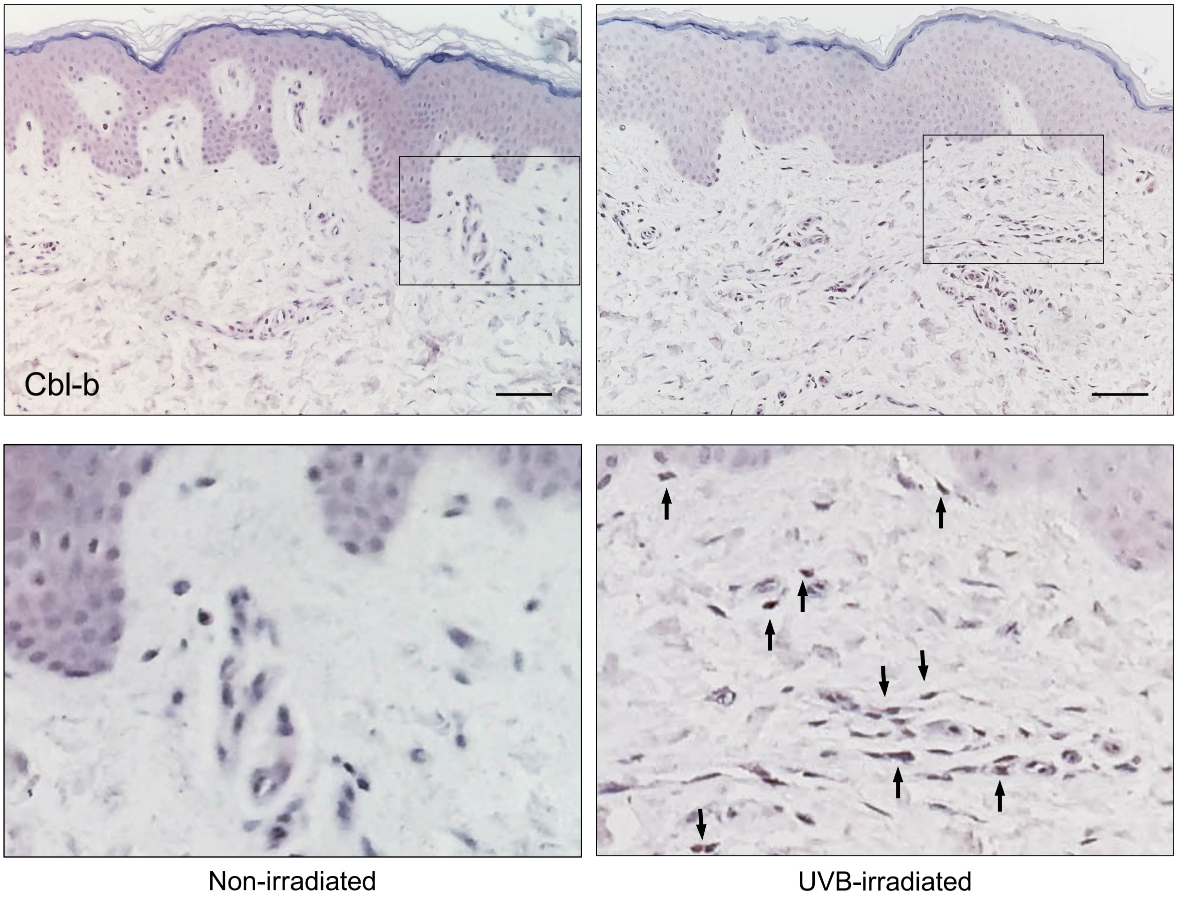

Supplementary figure 1: Paraffin sections of human skin 24h after UVB irradiation or non irradiated stained for Cbl-b. Marked squares of the upper panel are shown at higher magnification in the lower panel. Arrows indicate Cbl-b+ cells. Scale bar 50 µm.
