# Supplementary material for: Cbl-b deficiency provides protection against UVB-induced skin damage by modulating inflammatory gene signature
Source: Cell Death Dis. 2018 Aug 6;9(8):835. doi: 10.1038/s41419-018-0858-5 (PMC6079082; doi:10.1038/s41419-018-0858-5)
Supplement: Supplementary file 2 — Supplementary figure 2 [file 41419_2018_858_MOESM2_ESM.pdf]

Supplementary figure 2

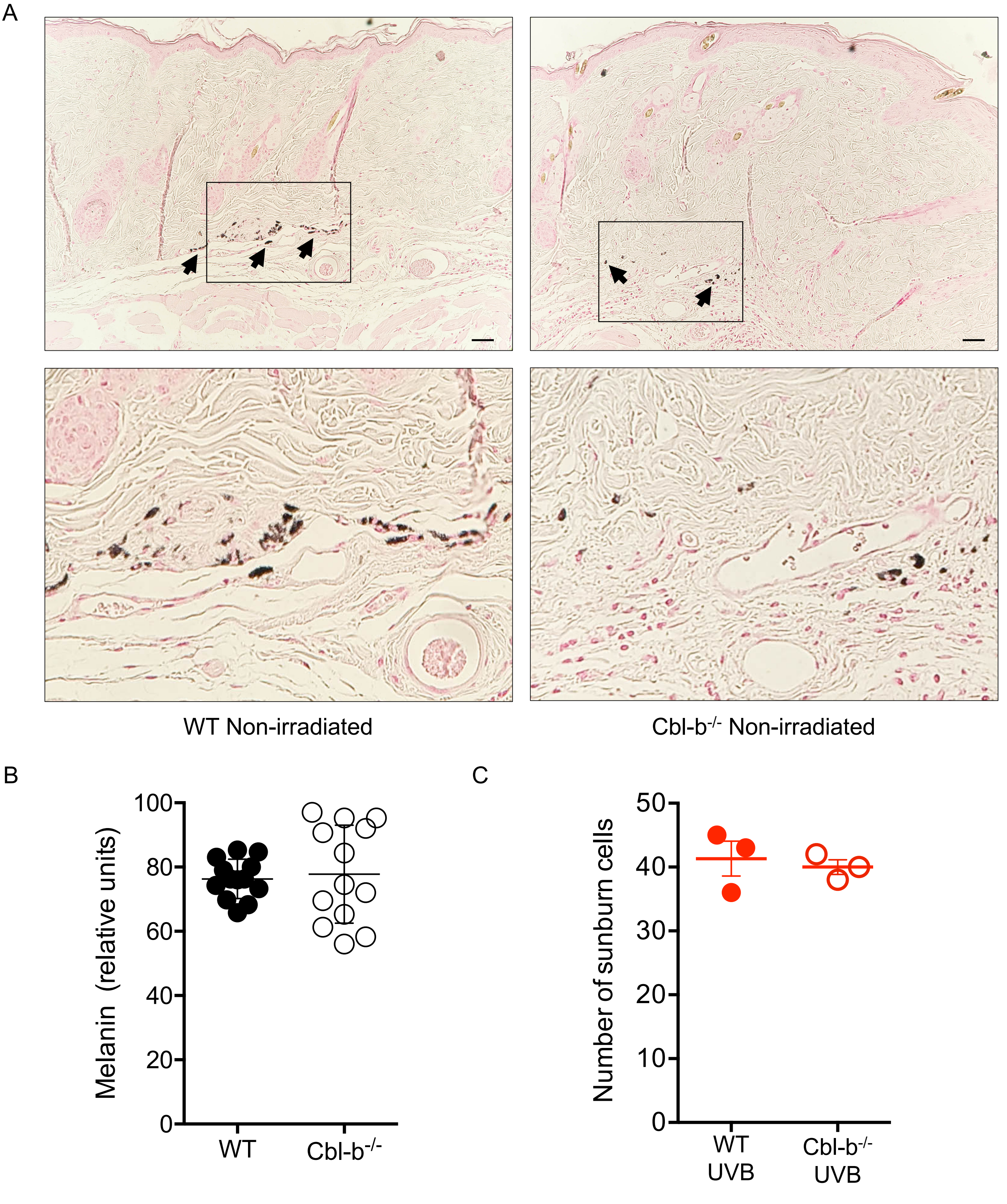

Supplementary figure 2: **(A)** Fontana-Masson stain of dorsal skin from non-irradiated WT and Cbl-b$^{-/-}$ mice. Marked squares of the upper panel are shown at higher magnification in the lower panel. Scale bar 50 µm. Arrows indicate melanin deposits. **(B)** Non invasive quantification of pigmentation (DermaSpectrometer, Cortex Technologies) of shaved dorsal skin of non-irradiated WT and Cbl-b$^{-/-}$ mice. **(C)** Number of SBCs at 6h after UVB irradiation.
