# Supplementary material for: Cbl-b deficiency provides protection against UVB-induced skin damage by modulating inflammatory gene signature
Source: Cell Death Dis. 2018 Aug 6;9(8):835. doi: 10.1038/s41419-018-0858-5 (PMC6079082; doi:10.1038/s41419-018-0858-5)
Supplement: Supplementary file 3 — Supplementary figure 3 [file 41419_2018_858_MOESM3_ESM.pdf]

A

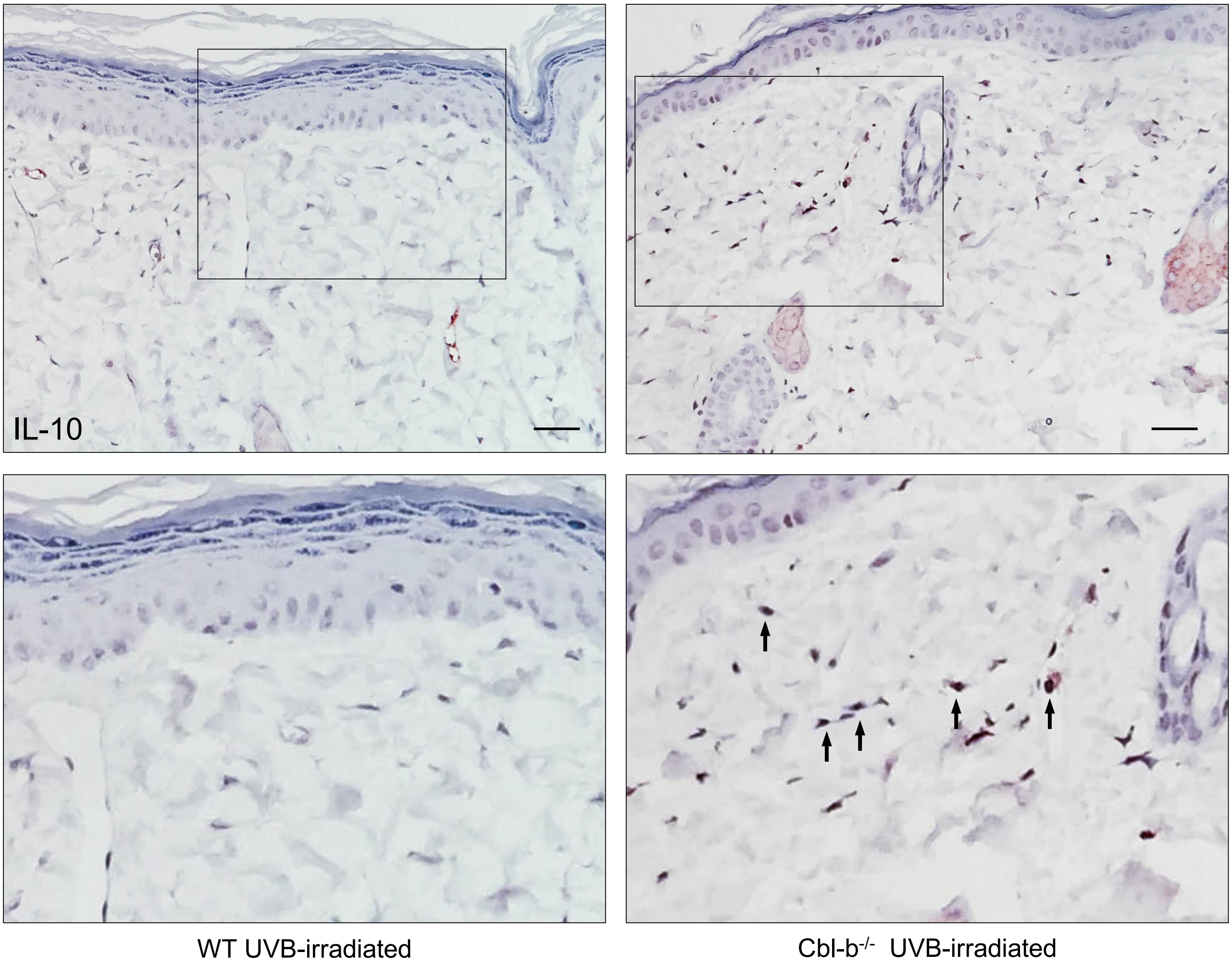

Supplementary figure 3: IL-10 stain of dorsal skin from UVB-irradiated WT and Cbl-b$^{-/-}$ mice. Marked squares of the upper panel are shown at higher magnification in the lower panel. Scale bar 50 µm. Arrows indicate IL-10+ cells.
