# Supplementary material for: Cbl-b deficiency provides protection against UVB-induced skin damage by modulating inflammatory gene signature
Source: Cell Death Dis. 2018 Aug 6;9(8):835. doi: 10.1038/s41419-018-0858-5 (PMC6079082; doi:10.1038/s41419-018-0858-5)
Supplement: Supplementary file 4 — Supplementary table 1: Gene co-expression analyses [file 41419_2018_858_MOESM4_ESM.pdf]

Supplementary table 1: Gene co-expression analyses

Categories	Function	Diseases or Functions Annotation	p-Value	z-score	No. Genes	Genes
**Cellular Movement, Immune Cell Trafficking**	**Migration**	**Leukocyte migration**	0.0021	**2.17**	9	**CBLB,Cxcl11,Defb8,F3,FLT4,MMP12,PROX1,RPL13A,TPSAB1/TPSB2**
Cardiovascular System Development and Function, Cellular Movement	Migration	Migration of endothelial cells	0.0174	**1.97**	4	F3,FLT4,NR4A1,PROX1
Cardiovascular System Development and Function, Cellular Development, Cellular Function and Maintenance, Cellular Growth and Proliferation, Organismal Development, Tissue Development	Proliferation	Proliferation of endothelial cells	0.0135	**1.95**	4	F3,FLT4,NR4A1,PROX1
Cellular Movement	Migration	Migration of cells	3E-05	**1.91**	18	CBLB,Cxcl11,CYGB,Defb8,Erdr1,F3,FLT4,FMOD,ITGB6,MMP12,NR1D1,NR4A1,PDCD4,PROX1,RPL13A,TIAM2,TPSAB1/TPSB2, WISP2
Cell-To-Cell Signaling and Interaction, Hematological System Development and Function	Activation	Activation of blood cells	0.0076	**1.72**	7	CBLB,CD300LB,F3,ITGB6,Saa3,SLC7A2,TPSAB1/TPSB2
Cellular Movement, Hematological System Development and Function, Immune Cell Trafficking	Cell movement	Cell movement of leukocytes	0.0121	**1.67**	7	CBLB,Cxcl11,Defb8,F3,MMP12,RPL13A, TPSAB1/TPSB2
Cardiovascular System Development and Function, Cellular Movement	Cell movement	Cell movement of endothelial cells	0.0044	**1.54**	5	F3,FLT4,NR4A1,OSR1,PROX1
Cardiovascular System Development and Function, Organismal Development	Vasculo-genesis	Vasculogenesis	0.0395	**1.51**	6	CYGB,F3,FLT4,MMP12,NR4A1,PROX1
Cellular Movement	Cell movement	Cell movement	8E-06	1.41	20	CBLB,Cxcl11,CYGB,Defb8,Erdr1,F3,FLT4,FMOD,ITGB6,MMP12,NR1D1,NR4A1,OSR1,PDCD4,PROX1,RPL13A,Saa3,TIAM2, TPSAB1/TPSB2,WISP2
Hematological System Development and Function, Immune Cell Trafficking, Inflammatory Response	Activation	Activation of leukocytes	0.019	1.41	6	CBLB,CD300LB,ITGB6,Saa3,SLC7A2, TPSAB1/TPSB2
Tissue Development	Growth	Growth of epithelial tissue	0.0068	1.30	7	CYP7B1,F3,FLT4,MMP12,NR4A1,OSR1, PROX1
Cellular Growth and Proliferation	Proliferation	Proliferation of cells	0.015	1.20	20	CBLB,CNTFR,CYGB,CYP7B1,Erdr1,F3,FLT4,FMOD,ITGB6,MMP12,NR1D1, NR4A1,OSR1, PDCD4, PROX1,TIAM2,TLR6,TPSAB1/TPSB2, VSIG4,WISP2
Cardiovascular System Development and Function, Cellular Movement	Movement	Movement of vascular endothelial cells	0.0016	1.19	4	FLT4,NR4A1,OSR1,PROX1
Carbohydrate Metabolism	Quantity	Quantity of carbohydrate	0.007	1.13	6	Gm15807/Hmgn5,NCEH1,NR4A1,PROX1, STEAP4,TPSAB1/TPSB2
Cell-To-Cell Signaling and Interaction	Activation	Activation of cells	0.0009	1.12	10	CBLB,CD300LB,F3,FMOD,ITGB6,NR4A1, Saa3,SLC7A2,TLR6,TPSAB1/TPSB2
Hematological System Development and Function, Inflammatory Response, Tissue Morphology	Quantity	Quantity of phagocytes	0.0113	1.09	5	F3,FLT4,ITGB6,MMP12,STEAP4
Hematological System Development and Function, Inflammatory Response, Tissue Morphology	Quantity	Quantity of macrophages	0.0029	1.07	4	FLT4,ITGB6,MMP12,STEAP4
Inflammatory Response	Inflammatory response	Inflammatory response	0.003	1.01	8	Cxcl11,Defb8,ITGB6,NR1D1,RPL13A,SLC7A2,TLR6,TPSAB1/TPSB2

Note: Ingenuity pathway analysis (IPA®) of upstream regulators (Ingenuity, QIAGEN-Bioinformatics) to infer the most likely activation state of biological processes (as indicated by categories and function). The association of genes is estimated by the z-score, as calculated by statistical modeling of published data.
